# Occurrence of post traumatic stress symptoms and their relationship to professional quality of life (ProQoL) in nursing staff at a forensic psychiatric security unit: a cross-sectional study

**DOI:** 10.1186/1477-7525-7-31

**Published:** 2009-04-16

**Authors:** Christian Lauvrud, Kåre Nonstad, Tom Palmstierna

**Affiliations:** 1 St. Olav's University Hospital, Division of Psychiatry, Forensic department Brøset, Centre for Research and Education in Forensic Psychiatry, Trondheim, Norway; 2 Dep. of Neuroscience, Faculty of Medicine NTNU, Trondheim, Norway; 3 Karolinska Institutet, Department of Clinical Neuroscience, Division of Forensic Psychiatry, Stockholm, Sweden

## Abstract

**Background:**

Violence is frequent towards nurses in forensic mental health hospitals. Implications of this high risk environment have not been systematically explored. This paper explores occurrence of symptoms on post traumatic stress and their relationship to professional quality of life.

**Methods:**

Self report questionnaires assessing symptoms of post traumatic stress and professional quality of life were distributed among psychiatric nurses in a high security forensic psychiatric unit with high frequency of violent behaviour. Relationships between post traumatic stress symptoms, forensic nursing experience, type of ward and compassion satisfaction, burnout and compassion fatigue were explored.

**Results:**

The prevalence of post traumatic stress symptoms was low. Low scores were found on compassion satisfaction. Length of psychiatric nursing experience and low scores on compassion satisfaction were correlated to increased post traumatic stress symptoms.

**Conclusion:**

Although high violence frequency, low rate of post traumatic stress symptoms and low compassion satisfaction scores was found. High staff/patient ratio and emotional distance between staff and patients are discussed as protective factors.

## Introduction

Psychiatric nurses often experience violence at their workplace. In the course of their career 70% experience violence against their person [1]. Being forced to manage violent patients often provokes adverse feelings and negative workplace experience [2]. This often causes feelings of fear and anxiety [3,4]. It has been shown that a substantial number of psychiatric nurses have signs of burnout. In a study of Robinson (2003) [5] more than 20% of registered psychiatric nurses reported intrusive memories from patient assaults. Recent findings indicate high level of emotional exhaustion among community mental health nurses, a phenomenon which seems to be lessened by regular clinical supervision [6]. Compassion fatigue and burnout is a phenomenon of importance for other health care and social workers and the relation between different aspects of job satisfaction, compassion fatigue and risk for burnout has been explored [7,8]. It has been argued the role conflict, a special feature in psychiatry caring as to the amount of violence nurses are forced to handle, that this role conflict in combination with low job satisfaction would promote burnout [9]. The role of violence directed towards nurses has already been shown to have a severe psychological impact on nurses afflicted [10]. Experiencing violence also increase the risk of more long term psychological consequences. The risk of having to leave nursing profession due to psychological consequences is substantial [11] and risk for post traumatic stress disorder (PTSD) after assaults against nurses is demonstrated high [12]. Since psychiatric units and especially forensic psychiatric units have a high degree of violence towards nurses it could be expected that burnout risks and symptoms of PTSD are prevalent among nurses in such institutions.

Interestingly in spite of being a high risk exposure environment the high security forensic psychiatry and the relationship between, and occurrence of job satisfaction, burnout and post traumatic stress symptoms has not been explored.

The aim of this present study is to explore relations between, and occurrence of, job satisfaction, burnout and post traumatic stress symptoms among nurses in high frequency violence environment.

## Methods

### Setting

The study was conducted in 2006 among nurses at Brøset regional secure unit. Brøset is a high secure forensic unit for severely mentally disordered patients too difficult to manage in acute and long-term psychiatric settings. Patients referred to Brøset are either sentenced to psychiatric care or referred from general psychiatric care units because of severe mental disorder or severe learning disabilities together with severe behaviour problems, mainly of a violent character. The patients referred to Brøset are considered too difficult to treat in the ordinary health care system. The majority of the patients are admitted on coercion. The unit serves regions in the central and northern parts of Norway with about 1.1 million inhabitants. The unit consists of 4 wards with a total number of 21 beds. One of the wards (ward F) is specialised to serve offenders with learning (intellectual) disabilities. The other three wards have different levels of control and structured environment in order to fit the needs of controlling the patients behaviour (ward A, admission ward with the highest structure, ward B intermediate and ward C with the least structure). This year the unit had a relatively high frequency of violence. During the year of the study (2006), staff experienced 221 incidents of threat and violence corresponding to 13.8 incidents/bed/year of which 7.4 incidents/bed/year were physical attacks on staff members. This frequency is higher than the average general acute ward where the overall frequency of violence in European countries is estimated to about 9.3 incidents/bed/year [13]. The higher frequency of violence at Brøset could be explained by the fact that patients admitted to Brøset are those with the most severe behavioural problems in the region.

### Assessments

Approximately 100 staff members were administered questionnaires regarding occurrence of post traumatic stress symptoms and their professional quality of life. For assessing post traumatic stress symptoms, the PTSD Checklist, civilian version (PCL-C) was used [14,15]. This is a 17- item self report measure developed to assess symptoms following the criteria for post traumatic stress disorder in the Diagnostic and Statistical Manual of Mental Disorders (DSM-IV). The different symptoms are answered by the respondent on a 5-point Likert scale to rate the extent to which they had been bothered in the past month by 17 symptoms of post traumatic stress based on the DSM-IV symptom clusters: reexperiencing, avoidance/numbing, and arousal. Weathers et al. [15] suggested that a symptom should be considered as meeting the threshold criterion if an individual reports that it has bothered him or her moderately, quite a bit, or extremely (i.e., an item endorsement of 3 or greater on the Likert scale).

Assessments of professional quality of life were made with the Professional Quality of Life Scale (ProQOL) [16], which is a validated development of the Compassion Fatigue Test [17]. ProQOL is a 30 item self-report measure to assess the dimensions compassion satisfaction, burn-out and compassion fatigue. The compassion satisfaction dimension (CS) measures pleasure derived from being able to do you work well where high scores represent a greater satisfaction related to your ability to be an effective caregiver. The burnout dimension (BO) in this scale is associated with feelings of hopelessness and difficulties in dealing with your work. Higher scores are related to higher risk for burnout. The compassion fatigue dimension (CF) relates to work-related secondary exposure to extremely stressful events. High scores indicate that you are exposed to frightening experiences at work.

The questionnaires were distributed anonymously, without data on gender or age in order to minimise bias in self reporting of symptoms possibly related to workplace situation.

The regional committee on research ethics was consulted and the committee considered this study not liable for formal approval as only staff members participated and the study was part of the hospitals internal actions to initiate steps to ensure quality practice. The committee found this study to be in no conflict with medical research ethics according to the Helsinki declaration. The study design was presented and approved by the hospitals employee representatives and personnel safety representatives.

### Participants

At Brøset there are 100 fulltime nursing position corresponding to a high patient-to-staff level of 1:5. 100 questionnaires were sent out to ordinary members of the nursing staff. 70 questionnaires were returned. No reminders were sent out. Of the 70 respondents, 33 (47.1%) had >12 years of nursing experience in psychiatry, 24 (34.2%) had 4–12 years of experience and only 13 (18.6%) had less than 4 years of experience. Among the respondents, the most experienced staff worked at the most highly structured ward, ward A (table 1). Of the respondents, only 7 had a position of < 50% of full time work at the unit. The respondents were evenly distributed over the three wards, 11 (15.7%) worked at the ward for patients with learning disabilities, 21 (30%) worked at the most highly and restricted ward, 16 (22.9%) at the least restricted ward and 22 (31.4%) worked at the ward with intermediate restrictive environment (see Table 1: Length of psychiatric nursing experience – Ward cross tabulation).

**Table 1:** Length of psychiatric nursing experience – Ward cross tabulation

	years	A	B	C	F	Total
Length of psychiatric nursing experience	0–3	2	4	5	2	13
		9.5%	18.2%	31.3%	18.2%	18,6%
	
	4–6	3	4	3	2	12
		14.3%	18.2%	18.8%	18.2%	17,1%
	
	7–9	3	5	0	0	8
		14.3%	22.7%	.0%	.0%	11,4%
	
	10–12	1	2	1	0	4
		4.8%	9.1%	6.3%	.0%	5,7%
	
	over 12	12	7	7	7	33
		57.1%	31.8%	43.8%	63.6%	47,1%

Total N		21	22	16	11	70

### Statistics

Occurrences of any PTSD symptom as rated with PCL-C were correlated to items in the ProQOL scale, ward and years of experience in forensic psychiatric care with a multiple logistic regression procedure. The Statistical Package for Social Sciences, SPSS 14.0 for Windows was used.

## Results

Sixty-seven of the 70 respondents (95.7%) met criterion A (exposure) according to the PTSD diagnosis in DSM-IV, reporting within the last 30 days i.e. either a.) exposed to real threats containing serious physical violence, b.) witnessed others exposed to serious physical violence (kicking, beatings e.g.) or c.) self being exposed to serious physical violence.

None of the respondents filled criterions for full PTSD diagnosis. Three (4.3%) reported at least one symptom occurring moderately (i.e. scoring at least 3 on the Likert scale) in each of cluster B, C and D (re-experience, avoidance, increased arousal). Seventeen (24.3%) reported at least one symptom occurring within either cluster B (re-experience), C (avoidance) or D (increased arousal).

Ward A's mean total sum of clusters B, C and D in the PCL-C, indicating overall stress symptoms, was 25.4 (95% CI 22.4–28.4), ward B; 19.7 (95% CI 18.3–21.1), ward C; 19.7 (95% CI 18.1–21.3), ward F; 21.7 (CI 95% 17.6–25.9), df = 3:66, F-value = 6.03, p = .001, thus indicating a higher rate of PTSD symptoms at ward A.

**Figure 1:**
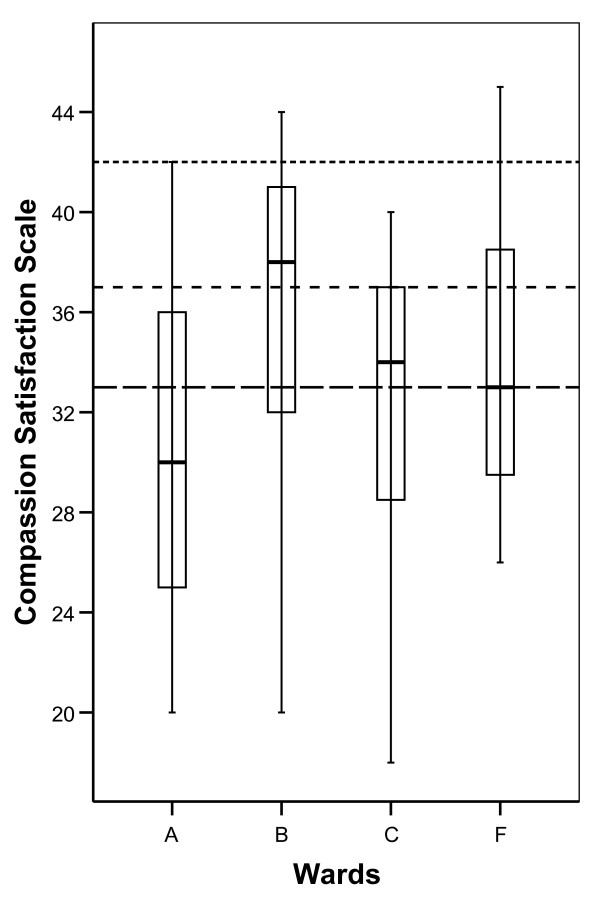
**Box plot indicating median, quartiles and extreme values for scores on CS at the different wards**. Horizontal dotted lines indicate bottom quartile, median and top quartile from normative data in the ProQOL manual [16].

Generally, compared to normative data, mean Compassion Satisfaction scores (CS) were low at all the wards. At ward A; mean CS was 30.2, (SD 6.5). Ward B; mean = 35.7, (SD 6.5). Ward C; mean = 31.5, (SD 8.3). Ward F; mean = 34 (SD 6.1) (See Figure 1).

**Figure 2:**
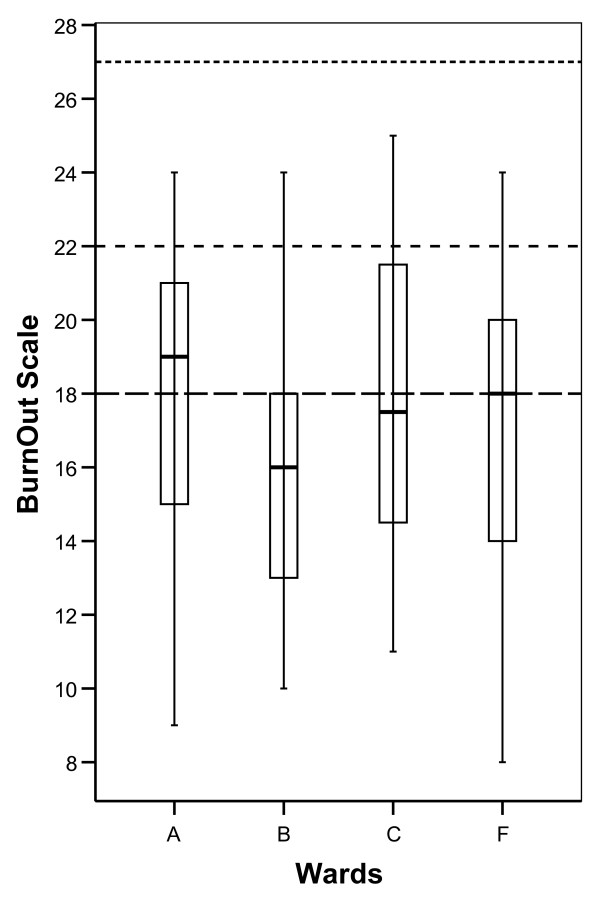
**Boxplot indicating median, quartiles and extreme values for scores on BO at the different wards**. Horizontal dotted lines indicate bottom quartile, median and top quartile from normative data in the ProQOL manual [16].

**Figure 3:**
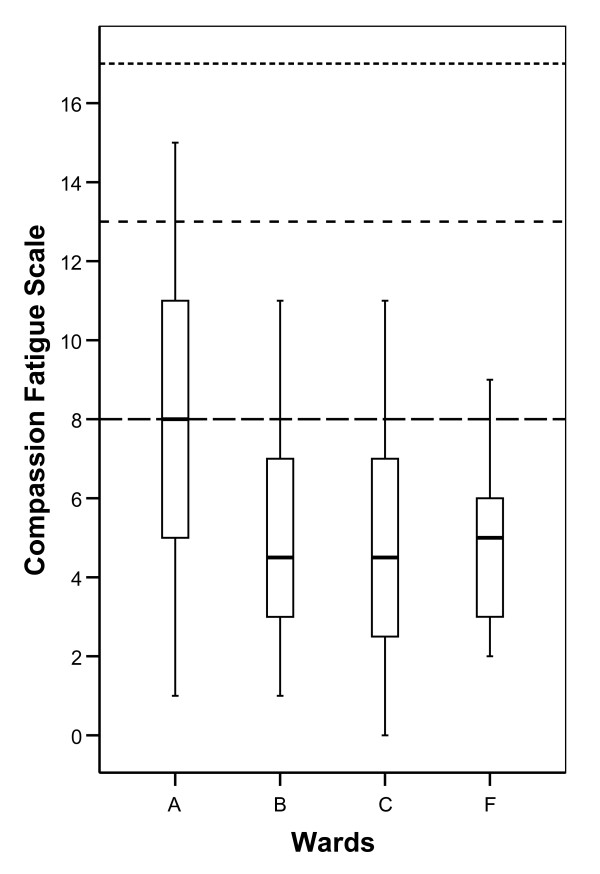
**Boxplot indicating median, quartiles and extreme values for scores on CF at the different wards**. Horizontal dotted lines indicate bottom quartile, median and top quartile from normative data in the ProQOL manual [16].

Burnout scale (BO) and Compassion Fatigue (CF) was in all four wards reported well below average scores according to normative data in the ProQOL manual (Figure 2 and Figure 3). Total mean BO was 17.3 (SD 4.4) and total mean CF was 5.8 (SD 3.6). In the boxplot Figure 1, Figure 2 and Figure 3, the separate distributions of scores for the wards of the ProQOL dimension are presented relative to normative data from the ProQOL manual [16] (see Figure 2 and Figure 3).

Occurrence of any symptoms of PTSD was related to the variables length of psychiatric nursing experience, which of the wards you were working at (as categorical variable) and scoring on the variables Compassion Satisfaction (CS), Burnout (BO) and Compassion Fatigue (CF) in the ProQOL in a binary logistic regression model using a forward stepwise method (Wald). In the final model, including only variables significantly contributing to the model, two variables were significant, length of psychiatric nursing experience (P = .028, HR = 1.76, CI 95% 1.06–2.90) and scores on the CS (P = .027, HR = .90, CI 95% .81– .99).

## Discussion and conclusion

In this investigation of nursing staff working in a high frequency violence psychiatric institution, a low prevalence of post traumatic stress symptoms is found in spite of high exposure to violence. A substantial number of respondents had some symptoms, but only few even met criteria for partial PTSD as defined by the International Consensus Group on depression and anxiety [18]. Our results seems substantially lower than those reported by Robinson (2003) [5] who reported 1.4% fulfilling PTSD criteria and 35% having any PTSD symptoms in a population of psychiatric nurses not selected from high security hospitals. Also, Richter (2006) [12] reported a high number of PTSD-syndrome (17%) in a population of recently assaulted nurses of which 11% persisted more than 6 months. It seems not reasonable to assume that the nurses in this investigation are less exposed to violence than the above mentioned studies since 80% report being assaulted and in total 95.7% met the exposure criteria (A) of the DSM-IV.

The low prevalence of PTSD symptoms among nursing staff at this unit could possibly be explained in several ways. A number of traumatized staff members could have had experienced symptoms of PSTD without being detected in this study because of its cross-sectional design. Some could have had decreased symptoms, some could have left work because of symptoms and among those not responding to the questionnaire, some nurses could have had more symptoms and therefore being reluctant to answer because of avoidance issues related to PTSD. But, if these findings of low prevalence of PTSD symptoms found in our study could be replicated with other designs, this low prevalence could perhaps be explained by the special characteristics of the Brøset clinic such as very high patient/staff ratio (1:5), which together with a generally strong collegiate spirit within the wards and a strong sense of mutual experience throughout the unit could contribute to the low frequency of PTSD symptoms. The fact that most of the staff is "still in the trenches" could also explain the low presence of PTSD symptoms. They have not been able to re-experience or avoid events as they regularly are exposed to new violent traumatic conflicts. The emotional distance between personnel and patients necessitated by a high security ward and a primarily behaviour based regime could possibly also reduce the emotional impact of violence towards nurses and other staff.

There are however differences between the wards where the admission ward (ward A) with the most restrictive environment and the most disturbed patients have the highest prevalence of PTSD symptoms. At the same time the best predictor of having any PTSD symptoms were long experience and low compassion satisfaction. The finding that long experience predicts symptoms is previously known. It could be speculated that experienced staff with PTSD symptoms tend to stay at a ward with high structure and less need for engagement in patients rehabilitation. The long experience of these nurses leads both to a higher exposure to violence, and therefore to higher rates of post traumatic problems, and to a "natural briefing" of the staff, so that nothing is unexpected, and therefore fewer things are potentially traumatizing.

One can also speculate on if one of the treatment principles, i.e. making the patient treatable at a lower security level can explain the low compassion satisfaction scores at ward A, the admission ward. At this particular forensic unit, patients transfer to a ward with reduced security level as soon as the patients are deemed receptive for treatment in a more non-restrictive environment. This may diminish staffs perception on completeness and a job well done.

From a career planning and manageable time perspective, the finding of low compassion satisfaction and length of experience in psychiatry predicting PTSD symptoms raise questions on for how long nurses should work fulltime in these high frequency violent environments. If these results are further corroborated, perhaps career planning over time should include reduced exposure to work with severely violent patients and perhaps offer other tasks duties instead, such as e.g. training and mentoring younger colleagues or like other highly violence exposed professions, acknowledge reduced retirement age such as is the case with police officers and firemen.

## Competing interests

The authors declare that they have no competing interests.

## Authors' contributions

CL, TP and KN conceived and designed the study. CL collected the data. CL and TP performed statistical analysis and drafted the manuscript. All authors revised the manuscript critically and approved the final manuscript.
